# Safety and Efficacy of Two Venous Closure Devices Compared With Manual Compression After Atrial Fibrillation Ablation

**DOI:** 10.1002/joa3.70442

**Published:** 2026-08-12

**Authors:** Satoko Shiomi, Kenichi Tokutake, Ui Takato, Ryutaro Sakurai, Yoshito Yamazaki, Takuya Matsumoto, Hidenori Sato, Hirotsuna Oseto, Masaaki Yokoyama, Seigo Yamashita, Teiichi Yamane, Michifumi Tokuda

**Affiliations:** ^1^ Department of Cardiology The Jikei University School of Medicine Tokyo Japan

**Keywords:** ablation, atrial fibrillation, complication, re‐bleeding, venous closure system

## Abstract

**Background:**

VASCADE MVP device has garnered attention as a novel femoral venous closure device. Manual compression (MC) is associated with prolonged bed rest and significant patient discomfort. Although Perclose ProGride/ProStyle device reduces bed rest duration, it can result in severe puncture site complications. The adoption of VASCADE MVP is anticipated to reduce the incidence of puncture site complications and facilitate early patient mobilization.

**Methods:**

A total of 858 patients undergoing catheter ablation were included in this study (MC: *n* = 196; Perclose: *n* = 547; VASCADE: *n* = 115). Perclose device was introduced in January 2018, and VASCADE device in September 2024. Venous closure devices were employed for all suitable femoral puncture sheaths. In the MC group, compression was released after 7 h or the following morning; in the Perclose group after 4 h and in the VASCADE group after three or 4 h. Total post‐procedure time and vascular complications were compared among the groups.

**Results:**

The total post‐procedure time was significantly shorter with VASCADE compared to Perclose and MC (22.6 ± 8.9 min vs. 29.6 ± 8.2 min and 30.4 ± 9.3 min, *p* < 0.001). The incidence of re‐bleeding was comparable between Perclose and VASCADE (11% vs. 13%, *p* = 0.787). No serious complications were observed in VASCADE group, whereas three patients (1%) in Perclose group experienced major complications.

**Conclusion:**

The use of VASCADE devices safely reduced both total post‐procedure time and bedrest times for complete hemostasis, without an associated increase in complications.

## Graphical abstract

In a cohort of 858 patients undergoing atrial fibrillation ablation, the use of the VASCADE device was associated with a significant reduction in both post‐procedural and bed‐rest durations compared to the Perclose device and manual compression. Although re‐bleeding rates were similar among the groups, no major vascular complications occurred with the VASCADE device, whereas the Perclose device was linked to rare but serious femoral vein complications. These findings highlight the favorable safety and efficacy profile of the VASCADE device.

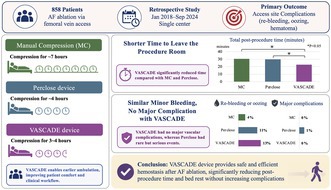

## 1 Introduction

Catheter ablation for atrial fibrillation necessitates the insertion of multiple large French (Fr) sheaths into femoral vein. Manual compression (MC) is the most common method for achieving hemostasis at the femoral access site; however, this technique requires prolonged bed rest following the procedure and is associated with considerable patient discomfort. The introduction of femoral venous closure devices has alleviated this burden by facilitating earlier postoperative ambulation. Perclose ProGride/ProStyle (Abbott, Lake Bluff, IL, USA) has demonstrated efficacy in reducing bed rest duration to approximately 4 h [1]. Nonetheless, its use has been linked to several serious complications, including femoral vein stenosis necessitating surgical intervention and difficulties with sheath removal. Consequently, there remains a need for a safer and more reliable femoral venous closure device. In this context, the introduction of VASCADE MVP (HAEMONETICS, Boston, MA, USA) offers a promising new alternative.

## 2 Methods

### 2.1 Study Population

A total of 858 patients who underwent atrial fibrillation (AF) ablation between January 2018 and September 2024 were included in this study (MC: *n* = 196; Perclose: *n* = 547; VASCADE: *n* = 115). AF ablation procedures were performed using radiofrequency, balloon devices (cryoballoon, laserballoon, and hotballoon), and pulsed‐field ablation. Paroxysmal AF was defined as AF that terminated spontaneously within 7 days. All patients received effective anticoagulation therapy for a minimum of 3 weeks prior to the procedure. On the day of ablation, Apixaban, Edoxaban, and Rivaroxaban were switched to Dabigatran, while Warfarin was continued with optimal control. During the procedure, the activated clotting time was maintained at greater than 300 s for radiofrequency or balloon devices and greater than 350 s for pulsed‐field ablation. Protamine 50 mg was administered at the conclusion of the procedure to reverse anticoagulation regardless of the final activated clotting time value.

All patients provided written informed consent regarding the treatment prior to the procedure. For this retrospective analysis, the Ethics Committee waived the requirement for additional written consent. Clinical investigations were conducted in accordance with the principles outlined in the Declaration of Helsinki. The study protocol and data collection procedures were approved by the Human Research Committee of the Jikei University School of Medicine (29‐361(8977)).

### 2.2 Puncture Site Hemostasis Method

All patients underwent two or three right femoral vein punctures, utilizing procedural sheaths with inner diameter from 5 to 13 Fr and outer diameter from 6 to 17 Fr. The deformable sheaths utilized in this study included Agilis NxT (Inner‐outer diameter: 8.5–11.5Fr; Abbott, St. Paul, MN), MobiCath (8.5–11.5Fr; Biosense Webster, Diamond Bar, CA, USA), Navigo (8.5–11.5Fr; J‐Sol Medical Co., Tokyo, Japan), VIZIGO (8.5–11.5Fr; Biosense Webster, Diamond Bar, CA, USA). For balloon ablation, FlexCath Advance (12–15Fr; Medtronic, Minneapolis, MN, USA) and POLARSHEATH (12.7–15.9Fr; Boston Scientific, Natick, MA, USA) were used for the cryoballoon, DG sheath (13–16Fr; TORAY, Tokyo, Japan) for the hotballoon, and laser balloon sheath (12–16Fr; Japan Lifeline Co., Tokyo, Japan) for the laserballoon. For pulsed‐field ablation catheters, VIZIGO, FlexCath Select (10–13Fr; Medtronic, Minneapolis, MN, USA), and FlexCath Contour (10–14Fr; Medtronic, Minneapolis, MN, USA) were employed. Additionally, SL0 (8.5–11.3Fr; Abbott, St. Paul, MN), 5Fr short, 7Fr short and 9 Fr long sheath (Terumo Co., Tokyo, Japan) were used as required.

A figure‐of‐eight suture was employed in cases managed with MC. Perclose device was introduced in March 2021, and VASCADE device was implemented beginning in January 2024. All venous closure procedures were performed in accordance with the manufacturer's recommended techniques. Following the introduction period for each device, only patients who underwent procedures successfully utilizing that specific device were included in the study cohort. The use of VASCADE device was restricted to sheaths with inner diameter 6–12 Fr. In the MC group, compression was released after 7 h or the following morning; in the Perclose group, after 4 h; in the VASCADE group, after 3 or 4 h. Prior literature has demonstrated that patients who underwent the Perclose procedure were ambulatory within 4 h following the intervention [2]. Cases in which venous closure systems failed were excluded from the analysis. Cases in which the venous closure device was ineffective and alternative procedures were required for patient management were excluded from the study. Re‐bleeding was defined as bleeding that necessitated additional compression. Prior to re‐compression, manual compression was performed in all cases to confirm the achievement of hemostasis. The duration of re‐compression was generally comparable to that of the initial compression, but it was not explicitly standardized across operators; it was excluded from the analysis.

The primary outcome was the incidence of site‐related major or minor complications, including re‐bleeding, oozing, and hematoma formation. Complications were classified as either minor or major according to previously established criteria [3]. Significant complications encompass vascular stenosis and substantial hemorrhage necessitating hospitalization.

The secondary outcome was the total post‐procedure time, defined as the interval between removal of the final sheath and the time at which the patient was able to leave the operating room. Prior to vacating the operating room, visual confirmation was obtained that hemostasis had been achieved, and the wound site was subsequently compressed and immobilized to maintain stability. A shorter post‐procedure time reflects improved workflow efficiency and a reduced burden on medical staff.

### 2.3 Statistical Analyses

Continuous variables were expressed as the mean ± standard deviation (SD). Comparisons of continuous variables were performed using an unpaired Student's *t*‐test or the Mann–Whitney *U* test, as appropriate. Categorical variables, presented as counts or proportions, were analyzed using the chi‐square test; when expected counts were less than 5, Fisher's exact test was employed. A *p* values of < 0.05 was considered statistically significant. For comparisons involving three or more groups, one‐way analysis of variance or the Kruskal–Wallis test was used, with the Tukey's or the Steel–Dwass post hoc test applied as appropriate. Non‐continuous variables were reported as median and interquartile range (IQR). Learning curves were evaluated using mixed‐effects models. A generalized linear mixed model and a linear mixed‐effects model using a binomial distribution and logit link function were applied. In both models, the cumulative experience of the operators (number of cases per operator) was treated as a fixed effect, and each operator was included as a random intercept to consider intra‐operator clustering. All statistical analyses were conducted using the SPSS software program (version 28.0.0.0; SPSS, Chicago, IL, USA).

## 3 Results

### 3.1 Patient Characteristics

Left atrial diameter was significantly larger in the MC group compared to the VASCADE group (39.9 ± 6.2 vs. 37.9 ± 6.0, *p* = 0.021). No significant differences were observed among the groups with respect to gender, body mass index (BMI), and left ventricular ejection fraction. Both the CHADS_2_ and HAS‐BLED scores were lower in the VASCADE group compared to the other two groups. The prevalence of comorbidities was not significantly different between groups, except for hypertension and dyslipidemia. Antiplatelet therapy was administered to 6% of patients in the MC group, 5% in the Perclose group, and 3% in VASCADE group, with no significant difference observed. Warfarin was used in 2% of patients in the MC group, 3% in the Perclose group, and 1% in the VASCADE group (Table 1).

**TABLE 1:** Patient background characteristics.

	MC group (*n* = 196)	Perclose group (*n* = 547)	VASCADE group (*n* = 115)	*p*
Gender (female)	32 (16%)	98 (18%)	22 (9%)	0.806
Age (y/o)	63.0 ± 9.6	61.9 ± 10.2	61.2 ± 11.2	0.260
Left atrium diameter (mm)	39.9 ± 6.2*	39.2 ± 6.5	37.9 ± 6.0*	0.029
Left ventricle ejection fraction (%)	61.9 ± 8.6	60.6 ± 8.8	60.0 ± 9.3	0.123
BMI	24.6 ± 3.3	24.5 ± 3.6	23.8 ± 3.2	0.106
Risk score [IQR]				
CHADS_2_ score	1 [0–1]	1 [0–1]	0 [0–1]	0.028
CHA_2_DS_2_‐VASC score	1 [1–2]	1 [0–2]	1 [0–2]	0.088
HAS‐BLED score	1 [0–2]	1 [0–2]	1 [0–1]	< 0.001
Type of Ablation				
Paroxysmal atrial fibrillation (%)	105 (54%)	308 (56%)	70 (61%)	0.455
Radiofrequency (%)	139 (71%)*^,†^	296 (54%)*^,‡^	28 (24%)^†,‡^	< 0.001
Balloon ablation (%)	57 (29%)*^,†^	239 (44%)*^,‡^	72 (63%)^†,‡^	< 0.001
Pulse field ablation (%)	0	12 (2%)*	15 (13%)*	< 0.001
Comorbidities				
Heart failure	16 (8%)	40 (7%)	7 (6%)	0.795
Hypertension	92 (47%)*	254 (46%)^†^	38 (33%)*^,†^	0.019
Diabetes	23 (12%)	72 (13%)	10 (9%)	0.331
Dyslipidemia	66 (34%)	212 (39%)*	30 (26%)*	0.020
Cerebral infarction	13 (7%)	35 (6%)	4 (3%)	0.307
Anticoagulant				
Apixaban	64 (33%)*	119 (22%)*	33 (29%)	0.011
Edoxaban	79 (40%)*^,†^	290 (53%)*	67 (58%)^†^	0.002
Rivaroxaban	37 (19%)*	102 (19%)^†^	11 (10%)*^,†^	0.014
Dabigatran	13 (7%)	19 (3%)	3 (3%)	0.192
Warfarin	3 (2%)	17 (3%)	1 (1%)	0.125
Baseline antiplatelet	11 (6%)	27 (5%)	4 (3%)	0.700
Aspirin	6 (3%)	16 (3%)	2 (2%)	0.758
Clopidogrel	3 (2%)	4 (1%)	0	0.328
Prasugrel	0	1 (0.002%)	1 (1%)	0.284
Others	2 (1%)	6 (1%)	1 (1%)	0.976

*Note:* The data are presented as the mean ± standard deviation, median (interquartile range), or *n* (%). Significance level: *p < 0.05, ^†^ *p* < 0.05, ^‡^ *p* < 0.05.

Abbreviation: BMI, body mass index.

### 3.2 Hemostasis Results

**FIGURE 1:**
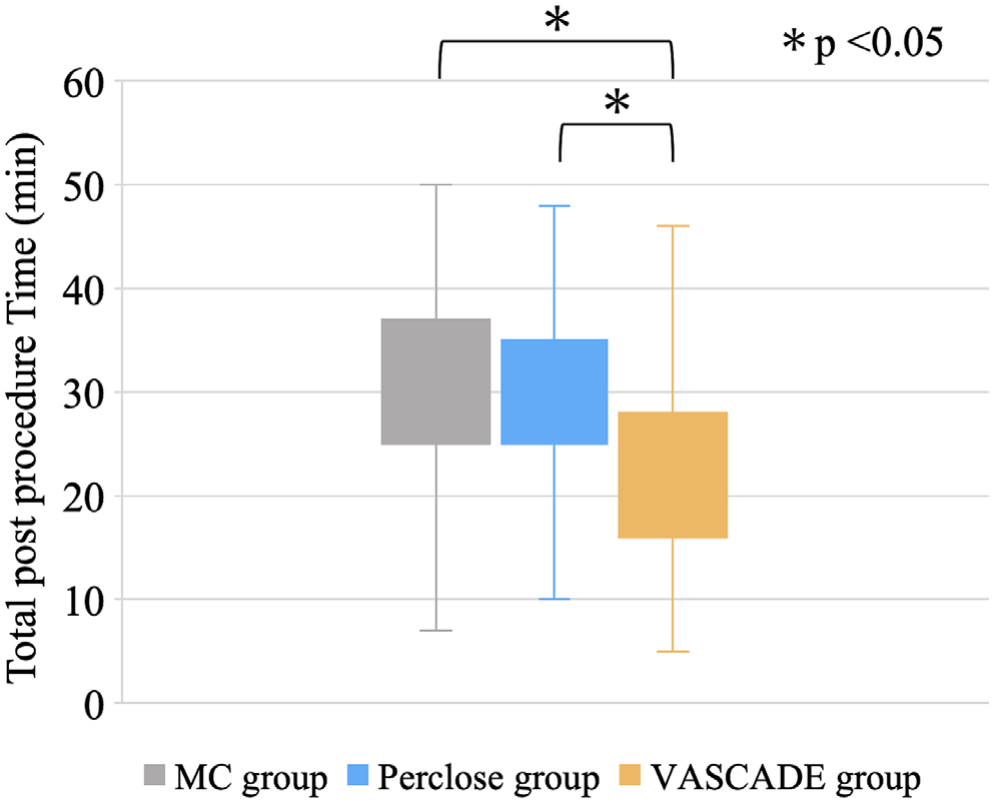
Box‐and‐whisker plot of total post‐procedure time. The interval between removal of the final procedural sheath and the patient's departure from the operating room was shortest in the VASCADE group. *p < 0.05.

Three sheaths were utilized in all cases within the MC group. The mean number of access sheaths in the VASCADE group was significantly lower than in other groups (VASCADE vs. MC: 2.8 ± 0.4 vs. 3, *p* < 0.001; VASCADE vs. Perclose: 2.8 ± 0.4 vs. 3.0 ± 0.1, *p* < 0.001). In the Perclose and VASCADE groups, two sheaths were used in 1% and 20% of cases, respectively (*p* < 0.001). The average outer diameter of femoral vein access sheath was largest in the VASCADE group and smallest in the MC group (*p* < 0.001). The maximum outer diameter of the femoral vein access sheath was also largest in the VASCADE group (*p* < 0.001). Sheaths with an inner diameter ≥ 12 Fr were used in 11% of MC cases and 23% of Perclose cases (*p* < 0.001) (Table 2). The total post‐procedure time was significantly shorter in the VASCADE group compared with both the Perclose and the MC groups (22.6 ± 8.9 min vs. 29.6 ± 8.2 min and 30.4 ± 9.3 min, *p* < 0.001) (Figure 1). In the linear mixed model analysis, the Perclose device exhibited a trend toward reduced total post‐procedure time with increasing operator experience; however, this association was not statistically significant (β = −0.042, *p* = 0.259). Similarly, for the VASCADE device, no significant association was observed between the number of experienced cases and hemostasis time (β = 0.053, *p* = 0.519). Procedural complications unrelated to access sites were observed in 5.6% (11/196) of the MC group, 2.6% (14/547) of the Perclose group, and 3.5% (4/115) of the VASCADE group, respectively. Complications included cardiac tamponade, retroperitoneal hematoma, pneumothorax, and symptomatic stroke.

**TABLE 2:** Procedural details of each group.

	MC group (*n* = 196)	Perclose group (*n* = 547)	VASCADE group (*n* = 115)	*p*
Max sheath size (Fr)	12.6 ± 1.9*^,†^	13.3 ± 2.1*^,‡^	13.9 ± 1.6^†,‡^	< 0.001
Average sheath outer diameter (Fr)	11.3 ± 0.6*^,†^	11.5 ± 0.9*^,‡^	11.9 ± 0.8^†,‡^	< 0.001
The number of access sites	3*	3.0 ± 0.1^†^	2.8 ± 0.4*^,†^	< 0.001
2 punctures	0	5 (1%)*	23 (20%)*	< 0.001
Total post procedure time	30.4 ± 9.3*	29.6 ± 8.2^†^	22.6 ± 8.9*^,†^	< 0.001
Complications				
Major complications	0	3 (1%)	0	—
Minor complications	2 (1%)	1 (0.2%)	0	0.787
Re‐bleeding or ooze	8 (4%)*^,†^	59 (11%)*	15 (13%)^†^	< 0.001
Hematoma	3 (2%)	1 (0.1%)	2 (2%)	0.159

*Note:* The data are presented as the mean ± standard deviation, median (interquartile range), or *n* (%). Significance level: *p < 0.05, ^†^ *p* < 0.05, ^‡^ *p* < 0.05.

### 3.3 Complications of the Access Site

The incidence of re‐bleeding was comparable between the Perclose and VASCADE groups (11% vs. 13%, *p* = 0.787) (Table 2). No significant association was observed between the operator's experience (cumulative cases performed) and rate of freedom from rebleeding for any of the devices (Perclose: *F* = 1.649, *p* = 0.200; VASCADE: *F* = 0.013, *p* = 0.909). In the VASCADE group, 57 participants (50%) completed their bed rest duration within 3 h. Within the VASCADE group, there was no significant difference in the re‐bleeding rate between patients who had compression released at three versus 4 h post‐procedure (12% vs. 14%, *p* = 0.812).

**FIGURE 2:**
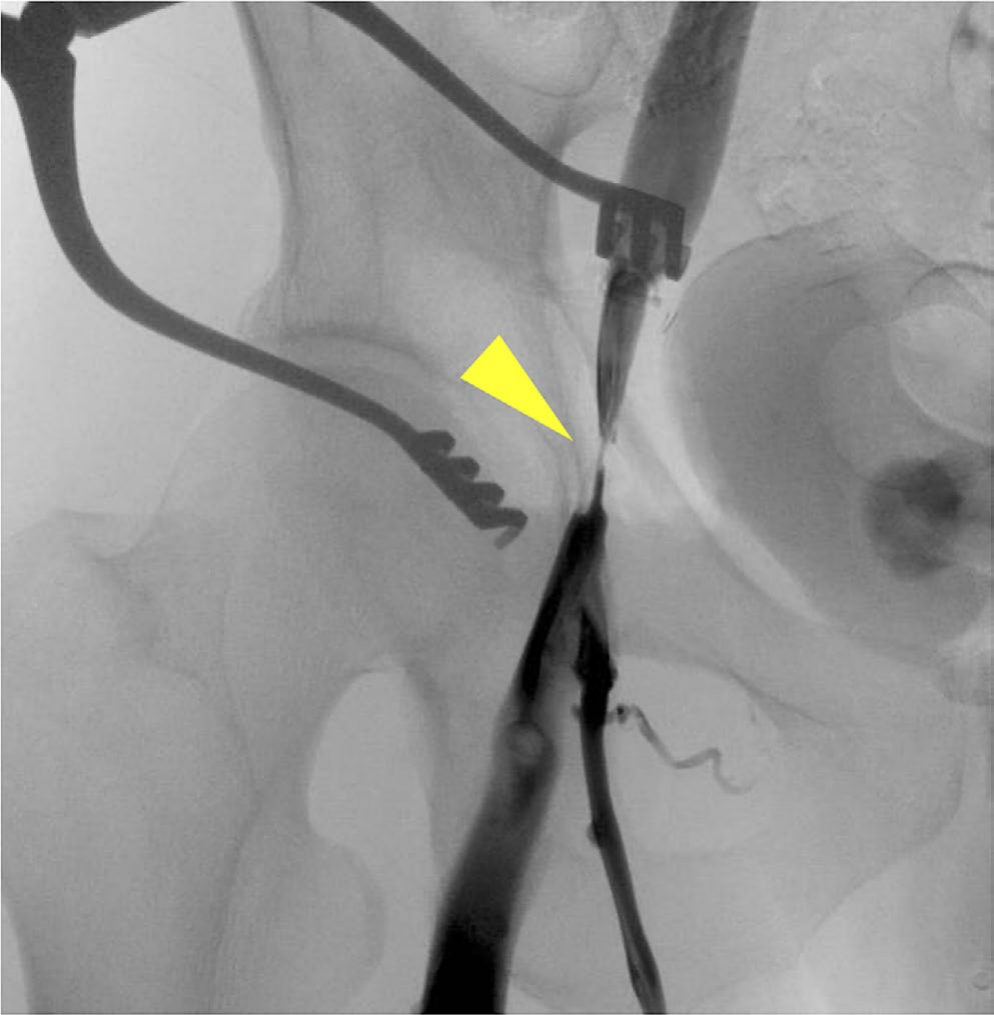
The stenosis of the right femoral vein due to Perclose. One week after surgery, the patient developed right lower leg edema. Contrast‐enhanced computed tomography revealed 99% stenosis of the femoral vein. Surgical exploration via femoral incision confirmed venous ligation caused by the Perclose device, which was subsequently released.

No serious complications occurred in either the VASCADE or MC groups. In contrast, serious complications were observed in three patients (1%) in the Perclose group. One patient developed femoral vein obstruction that required surgical patch plasty (Figure 2). In this case, three sheaths (16Fr, 9Fr, and 8.5Fr) were inserted into the right femoral vein. One week after the procedure, the patient presented with right lower extremity edema, and contrast‐enhanced computed tomography revealed 99% stenosis of the femoral vein. Surgical exploration confirmed Perclose‐induced ligation of the vein, which was subsequently released. Another case presented with right lower extremity edema that did not require surgical intervention. The third patient developed anemia and a large hematoma associated with severe pain, necessitating prolonged pain management. Notably, one operator encountered edema complications after using the Perclose device in more than 10 cases. Conversely, another operator observed vascular stenosis after using Perclose in over 140 cases, and a giant hematoma was noted after more than 30 cases.

### 3.4 Re‐Bleeding of VASCADE Device

**FIGURE 3:**
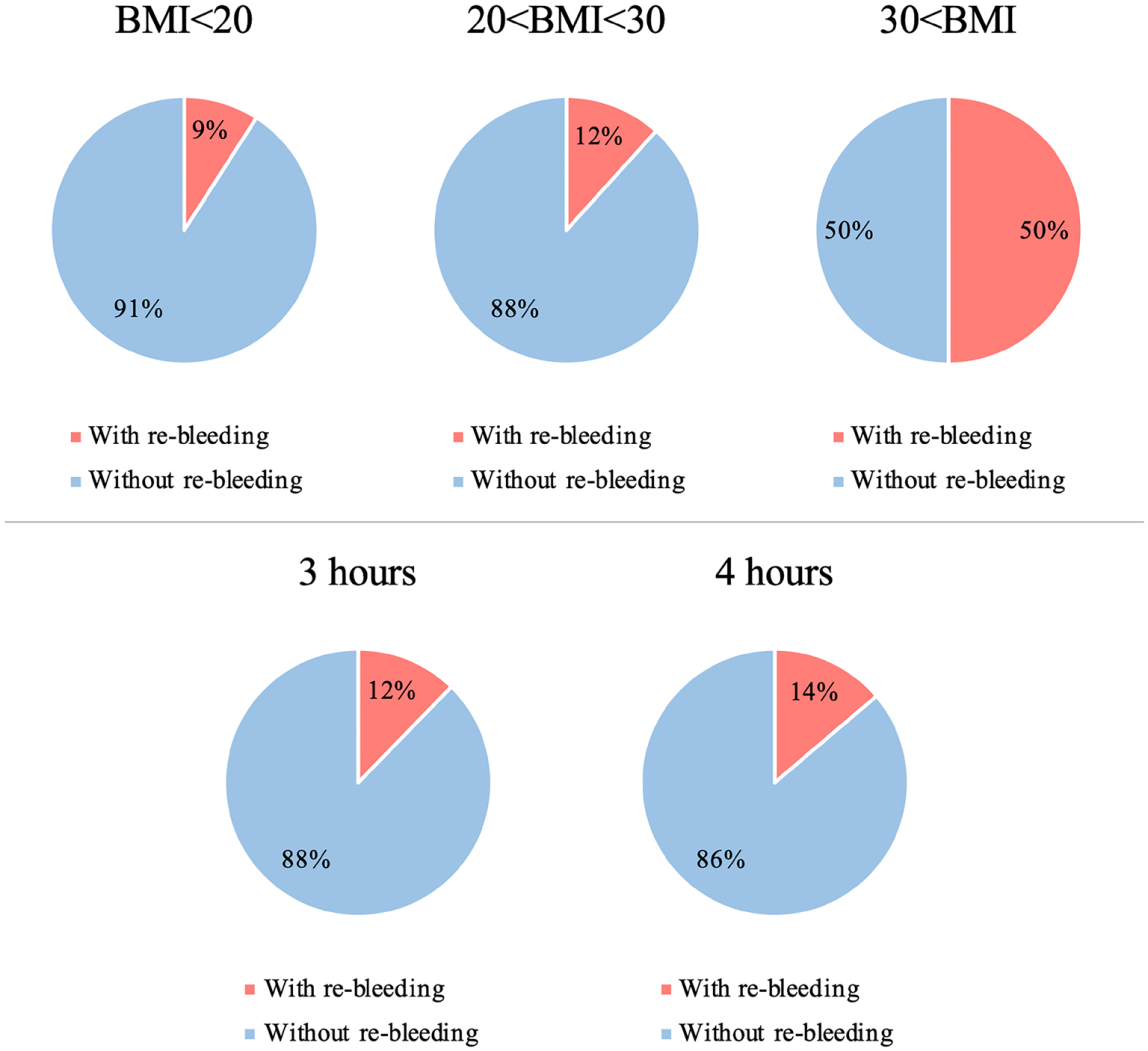
Pie chart of re‐bleeding rate by BMI and compression time. A BMI < 20 was not identified as a risk factor for re‐bleeding, whereas a BMI > 30 was associated with a significantly increased risk of re‐bleeding. Compression duration did not have a significant impact on the incidence of re‐bleeding. BMI, body mass index.

There were no cases of re‐bleeding that required blood transfusion or associated with the formation of large hematomas. All episodes of re‐bleeding were minor and resolved with the application of light manual pressure. Re‐bleeding occurred significantly more frequently in patients with BMI > 30 (BMI < 20: 9% vs. 20 ≦ BMI < 30: 11% and BMI > 30: 50%, *p* = 0.021). The duration of compression (3 vs. 4 h) did not influence the rate of re‐bleeding (12% vs. 14%, *p* = 0.812) (Figure 3). The number and size of sheathes, gender, and use of antiplatelet agents were not associated with the incidence of re‐bleeding.

## 4 Discussion

### 4.1 Main Findings

The use of VASCADE devices significantly reduced both total post‐procedural and bed rest duration without an associated increase in complications. No serious complications were observed, suggesting that the VASCADE devices may offer a favorable safety profile compared to other hemostatic methods.

### 4.2 VASCADE Device Compared to MC and Perclose Device

Ensuring safety is paramount when selecting venous closure systems, as effective hemostasis can be achieved with MC. MC is a well‐established hemostatic technique, and the findings of this study corroborate its efficacy, as evidenced by a notably low incidence of post‐procedural bleeding. Nevertheless, extended periods of bed rest exceeding 7 h have been associated with considerable patient discomfort. Recent reports indicate that Perclose is comparable to MC in terms of safety [4, 5] while also enabling earlier ambulation and reducing postoperative pain [6] In addition, although MC has occasionally resulted in major bleeding events, the incidence of such complications has decreased with the use of intravenous hemostatic devices.

Nevertheless, serious complications have been documented [7, 8]. Identified risk factors include obesity and inadequate sheath sizing techniques. In the case of Perclose devices, operator proficiency improves with experience, with the learning curve plateauing after 40 cases [9]. At our institution, vascular stenosis requiring surgical intervention was observed even when procedures were performed by experienced operators. Complications arising after otherwise successful catheter ablation procedures can impose a considerable burden on patients. Moreover, patients treated with Perclose device often report greater groin tenderness compared to those managed with MC [1]. The non‐absorbable nature of Perclose sutures, which remain in situ indefinitely, currently represents a contraindication for the use of VASCADE device and may pose challenges for the introduction of future venous closure devices.

### 4.3 Safe Use of VASCADE Device

VASCADE device has been reported to reduce postoperative rest time without causing serious complications [3]. Minor complications, including puncture site hematoma, pseudoaneurysm, and local infection, have each been observed in approximately 1% of cases [10, 11, 12].

The duration of post‐procedural rest did not influence the incidence of re‐bleeding, suggesting that 3 h of rest is sufficient. A BMI greater than 30 was identified as a clear risk factor for re‐bleeding. Caution is recommended when using VASCADE devices in patients with a BMI < 20 or BMI > 45. Notably, the rate of re‐bleeding did not increase among patients with low body weight, which may be attributed to the ability to compress and insert the collagen component subcutaneously, potentially enhancing the hemostatic effect. In contrast, obese patients (BMI > 30) require careful monitoring due to an elevated risk of re‐bleeding.

In this study, VASCADE devices were shown to reduce both bed rest duration and the time required to achieve post‐procedure hemostasis, without increasing the incidence of re‐bleeding compared to Perclose. Pulsed‐field catheter ablation has emerged as the predominant therapeutic modality for the management of atrial fibrillation, with a mean procedural duration of approximately 100 min [13]. Among these findings, the 8‐min reduction in the time required from achievement of hemostasis to patient discharge from the procedural room, representing approximately 8% of the total procedural duration, constitutes a clinically meaningful proportion of the overall intervention. This reduction in time lessens the burden not only on patients but also on medical staff. Furthermore, VASCADE device demonstrated a favorable safety profile, with no serious complications observed.

### 4.4 Study Limitations

Several limitations of the present study should be acknowledged. First, this was a retrospective, single‐center, and non‐randomized study. Due to the extended duration of the study, it was challenging to compare procedures performed by the same operator. Hemostatic procedures were carried out by multiple operators, which may have introduced variability and limited the standardization of techniques. The impact of learning or proficiency curves for each venous closure device cannot be excluded. Differences in puncture angle, puncture distance, and the amount of pressure applied immediately after final sheath removal may have influenced both complication rates and total procedure time. In addition, the introduction of new venous closure devices during the study period led to changes in hemostatic techniques, potentially resulting in group size imbalances and variations in data collection. Modifications in ablation catheters and sheaths over time may also have affected patient selection and clinical outcomes.

## 5 Conclusion

VASCADE device facilitated safe and efficient hemostasis, resulting in a significant reduction in both total post‐procedural and bed rest times without an associated increase in vascular complications.

## Funding

The authors have nothing to report.

## Disclosure

All authors take responsibility for all aspects of the reliability and freedom from bias of the data presented and their discussed interpretation.

## Ethics Statement

This study was performed in line with the principles of the Declaration of Helsinki. Approval was granted by the Ethics Committee of the Jikei University School of Medicine (29‐361(8977)).

## Consent

All patients provided written informed consent prior to the procedure.

## Conflicts of Interest

Michifumi Tokuda received consulting fee from Medtronic, research funding from Japan Lifeline Co. LTD, and Teiichi Yamane received speaker honoraria from Medtronic Japan, and research grants from Japan Lifeline Co. LTD. Other authors declare that they have no conflicts of interest.

## Data Availability

Data sharing not applicable to this article as no datasets were generated or analysed during the current study.

## References

[1] S. Kiani , J. Eggebeen , M. Al‐Gibbawi , et al., “Costs, Efficiency, and Patient‐Reported Outcomes Associated With Suture‐Mediated Percutaneous Closure for Atrial Fibrillation Ablation: Secondary Analysis of a Randomized Clinical Trial,” Journal of Cardiovascular Electrophysiology 35, no. 12 (2024): 2372–2381. 39377569 10.1111/jce.16440 PMC11650525

[2] A. Chikata , T. Kato , K. Usuda , et al., “Preclose Versus Postclose Using Suture‐Mediated Vascular Closure System for Catheter Ablation With Femoral Vein Access,” JACC Clinical Electrophysiology 10, no. 8 (2024): 1828–1836. 38795098 10.1016/j.jacep.2024.03.033

[3] A. Natale , S. Mohanty , P. Y. Liu , et al., “Venous Vascular Closure System Versus Manual Compression Following Multiple Access Electrophysiology Procedures: The AMBULATE Trial,” JACC: Clinical Electrophysiology 6, no. 1 (2020): 111–124. 31971899 10.1016/j.jacep.2019.08.013

[4] M. Mohammed , R. Ramirez , D. A. Steinhaus , et al., “Comparative Outcomes of Vascular Access Closure Methods Following Atrial Fibrillation/Flutter Catheter Ablation: Insights From VAscular Closure for Cardiac Ablation Registry,” Journal of Interventional Cardiac Electrophysiology 64, no. 2 (2022): 301–310. 33796968 10.1007/s10840-021-00981-5

[5] H. Lodhi , S. Shaukat , A. Mathews , B. Maini , and H. Khalili , “Comparison of Figure‐Of‐Eight Suture and Perclose ProGlide Suture‐Mediated Closure in Large Bore Venous Access Hemostasis: A Randomized Controlled Trial,” American Journal of Cardiology 209 (2023): 181–183. 37863115 10.1016/j.amjcard.2023.09.105

[6] D. Fabbricatore , D. Buytaert , C. Valeriano , et al., “Ambulatory Pulmonary Vein Isolation Workflow Using the Perclose ProglideTM Suture‐Mediated Vascular Closure Device: The PRO‐PVI Study,” Europace 25, no. 4 (2023): 1361–1368. 36793243 10.1093/europace/euad022 PMC10105833

[7] Y. Huang , X. Xie , and L. Wang , “Endovascular Perclose ProGlide Complication Puncture Site, Treated Successful by Cutting Balloon Dilatation: A Case Report and Literature Review,” Science Progress 107, no. 3 (2024): 368504241278481. 39279272 10.1177/00368504241278481 PMC11403694

[8] T. Ikenouchi , M. Takigawa , M. Goya , T. Kudo , and T. Sasano , “Refractory Deep Vein Thrombosis Caused by Femoral Vein Stenosis due to Suture‐Medicated Vascular Closure Device,” International Journal of Angiology 32, no. 4 (2023): 288–291. 10.1055/s-0042-1751230 PMC10624525 37927830

[9] E. Varis and D. C. Gurbuz , “Learning Curve of Perclose ProGlide Utilization During Percutaneous Coronary Intervention,” Cureus 15, no. 4 (2023): e38155. 37252468 10.7759/cureus.38155 PMC10217787

[10] E. B. Saad , A. Rossillo , C. P. Saad , et al., “Pulmonary Vein Stenosis After Radiofrequency Ablation of Atrial Fibrillation: Functional Characterization, Evolution, and Influence of the Ablation Strategy,” Circulation 108, no. 25 (2003): 3102–3107. 14623799 10.1161/01.CIR.0000104569.96907.7F

[11] A. Al‐Ahmad , S. Mittal , D. DeLurgio , et al., “Results From the Prospective, Multicenter AMBULATE‐CAP Trial: Reduced Use of Urinary Catheters and Protamine With Hemostasis via the Mid‐Bore Venous Vascular Closure System (VASCADE MVP) Following Multi‐Access Cardiac Ablation Procedures,” Journal of Cardiovascular Electrophysiology 32, no. 2 (2021): 191–199. 33270306 10.1111/jce.14828

[12] S. Nagpal , L. E. Scierka , Y. Castro‐Dominguez , et al., “Real‐World VASCADE Closure Device Versus Manual Compression Use and Outcomes in Patients With Severe Common Femoral Artery Disease,” Catheterization and Cardiovascular Interventions 100, no. 5 (2022): 776–784. 36129818 10.1002/ccd.30405

[13] V. Y. Reddy , S. R. Dukkipati , P. Neuzil , et al., “Pulsed Field Ablation of Paroxysmal Atrial Fibrillation: 1‐Year Outcomes of IMPULSE, PEFCAT, and PEFCAT II,” JACC: Clinical Electrophysiology 7, no. 5 (2021): 614–627. 33933412 10.1016/j.jacep.2021.02.014

